# “Stormy waters ahead”: global emergence of carbapenemases

**DOI:** 10.3389/fmicb.2013.00048

**Published:** 2013-03-14

**Authors:** Gopi Patel, Robert A. Bonomo

**Affiliations:** ^1^Department of Medicine, Mount Sinai School of MedicineNew York, NY, USA; ^2^Research Service, Louis Stokes Cleveland Department of Veterans Affairs Medical CenterCleveland, OH, USA; ^3^Division of Infectious Diseases and HIV Medicine, University Hospitals Case Medical CenterCleveland, OH, USA; ^4^Department of Medicine, Case Western Reserve School of MedicineCleveland, OH, USA; ^5^Department of Molecular Biology and Microbiology, Case Western Reserve School of MedicineCleveland, OH, USA; ^6^Department of Pharmacology, Case Western Reserve School of MedicineCleveland, OH, USA

**Keywords:** carbapenemases, NDM-1, KPC, OXA-48, metallo-β-lactamases, CHDL

## Abstract

Carbapenems, once considered the last line of defense against of serious infections with Enterobacteriaceae, are threatened with extinction. The increasing isolation of carbapenem-resistant Gram-negative pathogens is forcing practitioners to rely on uncertain alternatives. As little as 5 years ago, reports of carbapenem resistance in Enterobacteriaceae, common causes of both community and healthcare-associated infections, were sporadic and primarily limited to case reports, tertiary care centers, intensive care units, and outbreak settings. Carbapenem resistance mediated by β-lactamases, or carbapenemases, has become widespread and with the paucity of reliable antimicrobials available or in development, international focus has shifted to early detection and infection control. However, as reports of *Klebsiella pneumoniae* carbapenemases, New Delhi metallo-β-lactamase-1, and more recently OXA-48 (oxacillinase-48) become more common and with the conveniences of travel, the assumption that infections with highly resistant Gram-negative pathogens are limited to the infirmed and the heavily antibiotic and healthcare exposed are quickly being dispelled. Herein, we provide a status report describing the increasing challenges clinicians are facing and forecast the “stormy waters” ahead.

Carbapenems are potent and broad-spectrum β-lactam antibiotics traditionally reserved for the treatment of the most serious infections (El-Gamal and Oh, 2010). The emergence and dissemination of carbapenem-resistant Gram-negative pathogens including *Pseudomonas aeruginosa*, *Acinetobacter baumannii*, and Enterobacteriaceae is a significant contributor to patient morbidity and mortality (Patel et al., 2008; Schwaber et al., 2008; Lautenbach et al., 2009, 2010; Marchaim et al., 2011). Despite radical efforts in infection control (Schwaber et al., 2011) and improvements in rapid molecular diagnostics (Centers for Disease Control and Prevention, 2009; Nordmann et al., 2012c), carbapenem-resistant Gram-negative bacilli remain a formidable threat as few antimicrobial agents are reliably active and very little is expected to be available in the near future.

Clinicians hold that the increasing prevalence of extended-spectrum β-lactamases (ESBLs) among *Klebsiella pneumoniae* and *Escherichia coli* in the 1980s and 1990s contributed to the increased consumption of carbapenems. Experience implied that delayed administration of carbapenems in at-risk patients led to poor clinical outcomes (Paterson and Bonomo, 2005; Endimiani and Paterson, 2007). Thus, carbapenems (i.e., imipenem, meropenem, ertapenem, and doripenem) became vital tools in the treatment of healthcare-associated and severe community-acquired infections. Despite heavy reliance on these agents, carbapenem resistance in Enterobacteriaceae, common causes of both community and healthcare-associated infections, remained rare until the past decade.

Carbapenem resistance among Gram-negative bacteria results from one or more of the following mechanisms: (i) hyperproduction or derepression of Ambler class C β-lactamases (AmpC β-lactamases) or ESBLs (e.g., sulfhydryl variable (SHV), temoneira (TEM), cefotaxime (CTX-M) type β-lactamases) with loss or alteration in outer membrane porins; (ii) augmented drug efflux; (iii) alterations in penicillin binding proteins (PBPs); (iv) carbapenemase production (Patel and Bonomo, 2011). Carbapenemases belong to three molecular classes of β-lactamases, Ambler class A, B, and D (Ambler, 1980; Bush and Jacoby, 2010). Our aim is to provide a status report of the molecular diversity and epidemiology of carbapenemases as well as current and future therapeutics. The increasing public safety concerns associated with organisms harboring these enzymes has created significant turmoil. Regrettably, the situation is critical and our patients are in peril.

## AMBLER CLASS A CARBAPENEMASES

Few Ambler class A β-lactamases demonstrate carbapenem-hydrolyzing activity and, up until a decade ago, these were rarely recovered. Class A carbapenemases include: *K. pneumoniae* carbapenemase (KPC), Guiana extended-spectrum (GES), non-metallo-carbapenemase-A (Nmc-A)/imipenem-resistant (IMI), *Serratia marcescens* enzyme (SME), serratia fonticola carbapenemase (SFC), and BIC β-lactamases (**Table 1**; Walther-Rasmussen and Høiby, 2007). With the notable exception of KPCs, the clinical isolation of these types of carbapenemases is relatively limited.

**Table 1:** Class A carbapenemases*.

Enzyme	Year isolated or described	Organism(s)	Origin and geographic distribution	Location	Reference
Nmc-A	1990	*Enterobacter cloacae*	France, Argentina, USA	Chromosomal	Nordmann et al. (1993)
IMI-1	1984	*Enterobacter cloacae*	USA	Chromosomal	Rasmussen et al. (1996)
IMI-2	1999	*Enterobacter asburiae*, *Enterobacter cloacae*	USA^†^, China	Plasmid	Aubron et al. (2005), Yu et al. (2006)
SME-1	1982	*S. marcescens*	UK, USA	Chromosomal	Naas et al. (1994)
SME-2	1992	*S. marcescens*	USA, Canada, Switzerland	Chromosomal	Deshpande et al. (2006a), Poirel et al. (2007), Carrer et al. (2008)
SME-3	2003	*S. marcescens*	USA	Chromosomal	Queenan et al. (2006)
SFC-1	2003	*S. fonticola*	Portugal^†^	Chromosomal	Henriques et al. (2004)
GES-2	2000	*P. aeruginosa*	South Africa	Plasmid	Vourli et al. (2004)
GES-4	2002	*K. pneumoniae*	Japan	Plasmid	Wachino et al. (2004)
GES-5	2001	*K. pneumoniae*, *E. coli*, *P. aeruginosa*	Greece, Korea, worldwide	Plasmid	Jeong et al. (2005), Viau et al. (2012)
GES-6	2003	*K. pneumoniae*	Greece	Plasmid	Viau et al. (2012)
GES-11	2008	*Acinetobacter baumannii*	France	Plasmid	Moubareck et al. (2009)
GES-14	2010	*A. baumannii*	France	Plasmid	Bogaerts et al. (2010)
KPC-1^‡^	1996	*K. pneumoniae*	USA	Plasmid	Yigit et al. (2001)
KPC-2	1998	Enterobacteriaceae, *P. aeruginosa*, *Acinetobacter* spp.	USA and worldwide	Plasmid^§^	Yigit et al. (2001)
KPC-3	2000	Enterobacteriaceae, *Acinetobacter* spp.	USA and worldwide	Plasmid	Woodford et al. (2004)
KPC-4	2003	*Enterobacter cancerogenus*, *K. pneumoniae*, *Acinetobacter* spp.	Scotland, Puerto Rico	Plasmid	Palepou et al. (2005), Robledo et al. (2007)
KPC-5	2006	*P. aeruginosa*	Puerto Rico	Plasmid	Wolter et al. (2009)
KPC-6	2003	*K. pneumoniae*	Puerto Rico	Plasmid	Bartual et al. (2005), Robledo et al. (2008)
KPC-7	2007	*K. pneumoniae*	USA	Plasmid	Perez et al. (2010a)
KPC-8	2008	*K. pneumoniae*	Puerto Rico	Plasmid	Diancourt et al. (2010)
KPC-9	2009	*E. coli*	Israel	Plasmid	Grosso et al. (2011)
KPC-10	2009	*Acinetobacter* spp.	Puerto Rico	Plasmid	Robledo et al. (2010)
KPC-11	2009	*K. pneumoniae*	Greece	Unknown	Da Silva et al. (2004)
KPC-12	2010	*E. coli*	China	Unknown	
KPC-13	2010	*Enterobacter cloacae*	Thailand	Unknown	
BIC-1	2009	*P. fluorescens*	France^†^	Chromosomal	Girlich et al. (2010)

* Adapted from Walther-Rasmussen and Høiby (2007).

† Environmental isolates.

‡ KPC-1 was later found to be the same enzyme as KPC-2 (Higgins et al., 2012a).

§ Chromosomal expression of *bla*_KPC-2_ has been described in *P. aeruginosa* (Villegas et al., 2007).

Non-metallo-carbapenemase-A is a chromosomal carbapenemase originally isolated from *Enterobacter cloacae* in France (Nordmann et al., 1993). Currently, reports of this particular β-lactamase are still rare (Pottumarthy et al., 2003; Castanheira et al., 2008; Osterblad et al., 2012). IMI-1 was initially recovered from the chromosome of an *Enterobacter cloacae* isolate in the southwestern USA (Rasmussen et al., 1996). A variant of IMI-1, IMI-2, has been identified on plasmids isolated from environmental strains of *Enterobacter asburiae* in USA rivers (Aubron et al., 2005).

SME-1 (*S. marcescens* enzyme) was originally identified in an isolate of *S. marcescens* from a patient in London in 1982 (Yang et al., 1990). SME-2 and SME-3 were subsequently isolated in the USA, Canada, and Switzerland (Naas et al., 1994; Queenan et al., 2000, 2006; Deshpande et al., 2006b; Poirel et al., 2007; Carrer et al., 2008). Chromosomally encoded SME-type carbapenemases continue to be isolated at a low frequency in North America (Deshpande et al., 2006a,b; Fairfax et al., 2011; Mataseje et al., 2012). Both SFC-1 and BIC-1 are chromosomal serine carbapenemases recovered from environmental isolates. The former from a *S. fonticola* isolate in Portugal (Henriques et al., 2004) and the latter from *Pseudomonas fluorescens* isolates recovered from the Seine River (Girlich et al., 2010).

The GES-type β-lactamases are acquired β-lactamases recovered from *P. aeruginosa*, Enterobacteriaceae, and *A. baumannii* (Poirel et al., 2000a; Castanheira et al., 2004a). The genes encoding these β-lactamase have often, but not exclusively, been identified within class 1 integrons residing on transferable plasmids (Bonnin et al., 2013; Walther-Rasmussen and Høiby, 2007). GES-1 has a similar hydrolysis profile to other ESBLs, although they essentially spare monobactams. Several GES β-lactamases are described with six (i.e., GES-2, GES-4, GES-5, GES-6, GES-11, and GES-14), demonstrating detectable carbapenemase activity in the setting of amino acid substitutions at their active sites (specifically at residue 104 and 170; Walther-Rasmussen and Høiby, 2007; Kotsakis et al., 2010). These GES-type carbapenemases have been described in Europe, South Africa, Asia, and the Middle East (Poirel et al., 2002; Jeong et al., 2005; da Fonseca et al., 2007; Moubareck et al., 2009; Bonnin et al., 2011, 2013).

Currently, most carbapenem resistance among Enterobacteriaceae in the USA and Israel is attributed to plasmid-mediated expression of a KPC-type carbapenemase (Endimiani et al., 2009b; Nordmann et al., 2009; Gupta et al., 2011; Schwaber et al., 2011). KPC-producing Enterobacteriaceae are considered endemic to Greece along with other carbapenemases, specifically VIM-type [Verona integron-encoded metallo-β-lactamases (MBLs); Canton et al., 2012]. KPCs efficiently hydrolyze carbapenems as well as penicillins, cephalosporins, and aztreonam and are not overcome *in vitro* by clinically available β-lactamase inhibitors (i.e., clavulanic acid, sulbactam, tazobactam – in fact these are hydrolyzed). These enzymes have been identified in several genera of Enterobacteriaceae as well as *Pseudomonas* spp. and *A. baumannii* (Miriagou et al., 2003; Yigit et al., 2003; Bratu et al., 2005; Villegas et al., 2007; Cai et al., 2008; Rasheed et al., 2008; Tibbetts et al., 2008; Robledo et al., 2010; Mathers et al., 2011; Geffen et al., 2012).

Carbapenem resistance secondary to KPC production was first described in a *K. pneumoniae* recovered in North Carolina in 1996 (Yigit et al., 2001). To date 12 KPC subtypes (KPC-2 to KPC-13; Robledo et al., 2008; Kitchel et al., 2009a; Navon-Venezia et al., 2009; Wolter et al., 2009; Gregory et al., 2010) have been reported with the vast majority of analyzed isolates expressing either KPC-2 or KPC-3.

**FIGURE 1:**
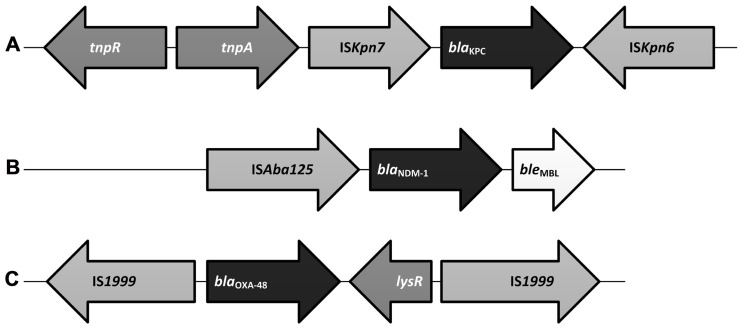
**FIGURE 1. Basic genetic construct of select carbapenemase genes. (A)** Schematic representation of Tn*4401* type of transposon associated with *bla*_KPC_ which includes a transposase gene (*tnp*A), a resolvase gene (*tnp*R), as well as insertion sequences, IS*Kpn6 *and IS*Kpn7* (Cuzon et al., 2010). **(B)** The *bla*_NDM-1_ construct demonstrates IS*Aba125* insertion sequence(s) upstream of the *bla*_NDM-1_ and a novel bleomycin resistance gene, *ble*_MBL_, downstream (Dortet et al., 2012). **(C)** *bla*_OXA-48_ is often mapped to a Tn*1999* composite transposon where it is bracketed between two copies of the same insertion sequence, IS*1999.* Downstream of *bla*_OXA-48_ lies a *lysR *gene which encodes for a regulatory protein (Poirel et al., 2012b).

The *bla*_KPC_ gene has been mapped to a highly conserved Tn*3*-based transposon, Tn*4401* (**Figure 1A**), and five isoforms of Tn*4401* are described (Naas et al., 2008; Cuzon et al., 2010; Kitchel et al., 2010). Plasmids carrying *bla*_KPC_ are of various sizes and many carry additional genes conferring resistance to fluoroquinolones and aminoglycosides thus limiting the antibiotics available to treat infections with KPC-producing pathogens (Endimiani et al., 2008; Rice et al., 2008). *bla*_KPC_ has rarely been mapped to a chromosomal location (Villegas et al., 2007; Castanheira et al., 2009).

A predominant strain of *K. pneumoniae* appears responsible for outbreaks and the international spread of KPC-producing *K. pneumoniae* (Woodford et al., 2008; Kitchel et al., 2009a; Samuelsen et al., 2009). Congruent pulsed-field gel electrophoresis (PFGE) patterns also suggest a clonal relationship between outbreak-associated strains of KPC-producing *K. pneumoniae* recovered from different areas that are endemic (Navon-Venezia et al., 2009; Woodford et al., 2011). The Centers for Disease Control and Prevention (CDC) performed PFGE and multilocus sequence typing (MLST) on isolates submitted to their reference laboratory from 1996 to 2008. A dominant PFGE pattern was observed and noted to be of a specific MLST type, ST 258 (Kitchel et al., 2009a). A second sequence type, ST 14, was common in institutions in the Midwest (Kitchel et al., 2009b). These findings implied that certain strains of *K. pneumoniae* may be more apt to obtain and retain the *bla*_KPC_ gene. Another study, however, analyzing 16 KPC-2 producing *K. pneumoniae* isolates from different geographic regions demonstrated diverse PFGE patterns and MLST types. This included four different MLST types in Colombia (ST 14, ST 337, ST 338, and ST 339) and two in Israel (ST 227 and ST 340). Although this study analyzed a smaller number of isolates, these findings suggest that the global propagation of KPC-2 is more complicated than the successful expansion of a fixed number of clones (Cuzon et al., 2010; Qi et al., 2011). More recently, a study evaluating the MLST types associated with widespread KPC-2 production in *K. pneumoniae* in Greece suggested that although ST 258 predominates at least 10 additional sequence types were found to carry *bla*_KPC-2_. Of note three (i.e., ST 147, ST 323, and ST 383) carried both KPC-2 as well as genes encoding VIM-type MBLs (Giakkoupi et al., 2011; Woodford et al., 2011). A retrospective study in Cleveland documented the presence of ST 36 in a long-term care facility for children (Viau et al., 2012).

*Klebsiella pneumoniae* carbapenemases-production can confer variable levels of carbapenem resistance with reported minimum inhibitory concentrations (MICs) ranging from susceptible to ≥ μg/mL. Analysis of isolates displaying high-level carbapenem resistance demonstrated that increased phenotypic resistance may be due to increased *bla*_KPC_ gene copy number or the loss of an outer membrane porin, OmpK35 and/or OmpK36. The highest level of imipenem resistance was seen with isolates lacking both porins and with augmented KPC enzyme production (Kitchel et al., 2010).

## AMBLER CLASS B CARBAPENEMASES: METALLO-β-LACTAMASES

Class B β-lactamases (**Table 2**) are referred to as MBLs and require a metal ion, usually zinc, for β-lactam hydrolysis (Walsh et al., 2005). Due to the dependence on Zn^2+^, catalysis is inhibited in the presence of metal-chelating agents like ethylenediaminetetraacetic acid (EDTA). MBL expression in Gram-negative bacteria confers resistance to penicillins, cephalosporins, and carbapenems. MBLs are not inhibited by the presence of commercially available β-lactamase inhibitors and susceptibility to monobactams (e.g., aztreonam) appears to be preserved in the absence of concomitant expression of other resistance mechanisms (e.g., ESBL production). The more geographically widespread MBLs include IMP (imipenem-resistant), VIM, and New Delhi metallo-β-lactamase (NDM).

**Table 2:** Metallo-β-lactamases.

Enzyme	Year isolated or described	Organism(s)	Geographic distribution	Location	Reference
IMP-1 to IMP-42	1988	Enterobacteriaceae, *Pseudomonas* spp., *Acinetobacter* spp.	Worldwide	Plasmid or chromosomal	Osano et al. (1994), Riccio et al. (2000)
VIM-1 to VIM-37	1997	Enterobacteriaceae, *Pseudomonas* spp., *Acinetobacter* spp.	Worldwide	Plasmid or chromosomal	Lauretti et al. (1999), Poirel et al. (2000b)
SPM-1	2001	*P. aeruginosa*	Brazil*	Chromosomal	Toleman et al. (2002)
GIM-1	2002	*P. aeruginosa*	Germany	Plasmid	Castanheira et al. (2004b)
SIM-1	2003–2004	*A. baumannii*	Korea	Chromosomal	Lee et al. (2005)
NDM-1 to NDM-7	2006	Enterobacteriaceae, *Acinetobacter *spp., *Vibrio cholerae*	Worldwide	Plasmid or chromosomal	Yong et al. (2009), Kaase et al. (2011), Nordmann et al. (2012a)
AIM-1	2007	*P. aeruginosa*	Australia	Chromosomal	Yong et al. (2007)
KHM-1	1997	*C. freundii*	Japan	Plasmid	Sekiguchi et al. (2008)
DIM-1	2007	*P. stutzeri*	Netherlands	Plasmid	Poirel et al. (2010c)
SMB-1	2010	*S. marcescens*	Japan	Chromosomal	Wachino et al. (2011)
TMB-1	2011	*Achromobacter xylosoxidans*	Libya	Chromosomal	El Salabi et al. (2012)
FIM-1	2007	*P. aeruginosa*	Italy	Chromosomal	Pollini et al. (2012)

* Single report of SPM-1 in Europe linked to healthcare exposure in Brazil (Salabi et al., 2010).

Chromosomal MBLs were the first to be identified and are the cause of carbapenem resistance observed in *Bacillus cereus*, *Aeromonas* spp., and *Stenotrophomonas maltophilia* (Walsh et al., 2005). However, of growing concern are the “mobile” MBLs that have been reported since the mid-1990s. Although most frequently found in carbapenem-resistant isolates of *P. aeruginosa* and occasionally *Acinetobacter* spp., there is growing isolation of these enzymes in Enterobacteriaceae.

Prior to the description of NDM-1, frequently detected MBLs include IMP-type and VIM-type with VIM-2 being the most prevalent. These MBLs are embedded within a variety of genetic structures, most commonly integrons. When these integrons are associated with transposons or plasmids they can readily be transferred between species.

In 1991, IMP-1, a plasmid-mediated MBL, was identified in an isolates of *S. marcescens* from Japan (Ito et al., 1995). Since then plasmid-mediated carbapenem resistance secondary to IMP-1 spread widely in Japan, Europe, Brazil, and other parts of Asia and in several species of Gram-negative bacilli including *Acinetobacter* spp. and Enterobacteriaceae. At the present time, 42 variants of IMP have been identified with most cases of IMP-mediated carbapenem resistance being reported from Asia and among *P. aeruginosa* (Bush and Jacoby, 2010).

A more commonly recovered MBL is the VIM-type enzyme. VIM-1 was first described in Italy in 1997 in *P. aeruginosa* (Lauretti et al., 1999). VIM-2 was next discovered in southern France in *P. aeruginosa* cultured from a neutropenic patient in 1996 (Poirel et al., 2000b). Although originally thought to be limited to non-fermenting Gram-negative bacilli, VIM-type MBLs are being increasingly identified in Enterobacteriaceae as well (Giakkoupi et al., 2003; Kassis-Chikhani et al., 2006; Morfin-Otero et al., 2009; Canton et al., 2012). To date, 37 variants of VIM have been described with VIM-2 being the most common MBL recovered worldwide.

Other more geographically restricted MBLs include SPM-1 (Sao Paulo MBL), which has been associated with hospital outbreaks in Brazil (Toleman et al., 2002; Rossi, 2011); GIM-1 (German imipenemase) isolated in carbapenem-resistant *P. aeruginosa* isolates in Germany (Castanheira et al., 2004b); SIM-1 (Seoul imipenemase) isolated from *A. baumannii* isolates in Korea (Lee et al., 2005); KHM-1 (Kyorin Health Science MBL) isolated from a *C. freundii* isolate in Japan (Sekiguchi et al., 2008); AIM-1 (Australian imipenemase) isolated from *P. aeruginosa* in Australia (Yong et al., 2007); DIM-1 (Dutch imipenemase) isolated from a clinical *P. stutzeri* isolate in the Netherlands (Poirel et al., 2010c); SMB-1 (*S. marcescens* MBL) in *S. marcescens* in Japan (Wachino et al., 2011); TMB-1 (Tripoli MBL) in *Achromobacter xylosoxidans* in Libya (El Salabi et al., 2012), and FIM-1 (Florence imipenemase) from a clinical isolate of *P. aeruginosa* in Italy (Pollini et al., 2012). With the notable exception of SPM-1, these MBLs have remained confined to their countries of origin (Salabi et al., 2010).

NDM-1 was first identified in 2008. Due to its rapid international dissemination and its ability to be expressed by numerous Gram-negative pathogens, NDM is poised to become the most commonly isolated and distributed carbapenemase worldwide. Initial reports frequently demonstrated an epidemiologic link to the Indian subcontinent where these MBLs are endemic (Kumarasamy et al., 2010). Indeed, retrospective analyses of stored isolates suggest that NDM-1 may have been circulating in the subcontinent as early as 2006 (Castanheira et al., 2011). Despite initial controversy, the Balkans may be another area of endemicity for NDM-1 (Struelens et al., 2010; Jovcic et al., 2011; Livermore et al., 2011c; Halaby et al., 2012). Sporadic recovery of NDM-1 in the Middle East suggests that this region may be an additional reservoir (Poirel et al., 2010a, 2011d; Nordmann et al., 2011; Ghazawi et al., 2012).

Like KPCs, the conveniences of international travel and medical tourism have quickly propelled this relatively novel MBL into a formidable public health threat. Gram-negative bacilli harboring *bla*_NDM_ have been identified worldwide with the exception of Central and South America.

NDM-1 was first identified in Sweden in a patient of Indian descent previously hospitalized in India (Yong et al., 2009). The patient was colonized with a *K. pneumoniae* and an *E. coli* carrying *bla*_NDM-1_ on transferable plasmids. In the UK, an increase in the number of clinical isolates of carbapenem-resistant Enterobacteriaceae was seen in both 2008 and 2009. A UK reference laboratory reported that at least 17 of 29 patients found to be harboring NDM-1 expressing Enterobacteriaceae had a history of recent travel to the Indian subcontinent with the majority having been hospitalized in those countries (Kumarasamy et al., 2010).

European reports suggest that horizontal transfer of *bla*_NDM-1_ exists within hospitals outside endemic areas. Of overwhelming concern are the reported cases without specific contact with the healthcare system locally or in endemic areas suggesting autochthonous acquisition (Kumarasamy et al., 2010; Kus et al., 2011; Arpin et al., 2012; Borgia et al., 2012; Nordmann et al., 2012b).

Surveillance of public water supplies in India indicates that exposure to NDM-1 may be environmental. Walsh et al. (2011) analyzed samples of public tap water and seepage water from sites around New Delhi. The results were disheartening in that *bla*_NDM-1_ was detected by PCR in 4% of drinking water samples and 30% of seepage samples. In this survey, carriage of *bla*_NDM-1_ was noted in 11 species of bacteria not previously described, including virulent ones like *Shigella boydii* and *Vibrio cholerae*.

The rapid spread of NDM-1 highlights the fluidity and rapidity of gene transfer between bacterial species. Although *bla*_NDM-1_ was initially and repeatedly mapped to plasmids isolated from carbapenem-resistant *E. coli* and *K. pneumoniae*, reports of both plasmid and chromosomal expression of *bla*_NDM-1_ has been noted in other species of Enterobacteriaceae as well as *Acinetobacter* spp. and *P. aeruginosa* (Moubareck et al., 2009; Bogaerts et al., 2010; Bonnin et al., 2011; Nordmann et al., 2011; Patel and Bonomo, 2011). Recently, bacteremia with a NDM-1 expressing *V. cholerae* has been described in a patient previously hospitalized in India colonized with a variety of Enterobacteriaceae previously known to be capable of carrying plasmids with *bla*_NDM-1_ (Darley et al., 2012).

In contrast to KPCs, the presence of a dominant clone among *bla*_NDM-1_ carrying isolates remains elusive (Poirel et al., 2011c). NDM-1 expression in *E. coli* has been noted among sequence types previously associated with the successful dissemination of other β-lactamases including ST 101 and ST 131 (Mushtaq et al., 2011). Mushtaq et al. (2011) analyzed a relatively large group of *bla*_NDM-1_ expressing *E. coli* from the UK, Pakistan, and India in order to potentially identify a predominant strain responsible for the rapid and successful spread of NDM-1. The most frequent sequence type identified was ST 101. Another study examining a collection of carbapenem-resistant Enterobacteriaceae from India demonstrates the diversity of strains capable of harboring *bla*_NDM-1_. Carriage of *bla*_NDM-1_ was confirmed in 10 different sequence types of *K. pneumoniae* and 5 sequence types of *E. coli* (Lascols et al., 2011). This multiplicity was confirmed in a study looking at a collection of *bla*_NDM-1_ expressing Enterobacteriaceae from around the world (Poirel et al., 2011c). Of most concern is that NDM-1 has been identified in *E. coli* ST 131, the strain of *E. coli* credited with the global propagation of CTX-M-15 ESBLs (Mushtaq et al., 2011; Peirano et al., 2011; Pfeifer et al., 2011b; Woodford et al., 2011). Similar to KPCs, NDM-1 expression portends variable levels of carbapenem resistance and there is often concomitant carriage of a myriad of resistance determinants including other β-lactamases and carbapenemases as well as genes associated with resistance to fluoroquinolones and aminoglycosides (Nordmann et al., 2011).

NDM-1 shares the most homology with VIM-1 and VIM-2. It is a 28-kDa monomeric protein that demonstrates tight binding to both penicillins and cephalosporins (Zhang and Hao, 2011). Binding to carbapenems does not appear to be as strong as other MBLs, but hydrolysis rates appear to be similar. Using ampicillin as a substrate, allowed for detailed characterization of the interactions between NDM's active site and β-lactams as well as improved evaluation of MBLs unique mechanism of β-lactam hydrolysis. More recent crystal structures of NDM-1 reveal the molecular details of how carbapenem antibiotics are recognized by dizinc-containing MBLs (King et al., 2012).

To date, NDM-1 remains the most common NDM variant isolated. Seven variants (NDM-1 to NDM-7) exist (Kaase et al., 2011; Nordmann et al., 2012a). It is currently held that *bla*_NDM-1_ is a chimeric gene that may have evolved from *A. baumannii* (Toleman et al., 2012). Contributing to this theory is the presence of complete or variations of the insertion sequence, IS*Aba125*, upstream to the *bla*_NDM__-1_ gene in both Enterobacteriaceae and *A. baumannii* (Pfeifer et al., 2011a; Poirel et al., 2011a; Dortet et al., 2012; Toleman et al., 2012). This insertion sequence has primarily been found in *A. baumannii*.

A recent evaluation of the genetic construct associated with *bla*_NDM-1_ (**Figure 1B**) has lead to the discovery of a new bleomycin resistance protein, BRP_MBL_. Evaluation of 23 isolates of *bla*_NDM-1__/__2_ harboring Enterobacteriaceae and *A. baumannii* noted that the overwhelming majority of them possessed a novel bleomycin resistance gene, *ble*_MBL_ (Dortet et al., 2012). Co-expression of *bla*_NDM-1_ and *ble*_MBL_ appear to be mediated by a common promoter (*P*_NDM-1_) which includes portions of IS*Aba125*. It is postulated that BRP_MBL_ expression may contribute some sort of selective advantage allowing NDM-1 to persist in the environment.

A contemporary evaluation of recently recovered NDM-1 producing *A. baumannii* isolates from Europe demonstrates that *bla*_NDM-1_ and *bla*_NDM-2_ genes are situated on the same chromosomally located transposon, Tn*125* (Bonnin et al., 2012). Dissemination of *bla*_NDM_ in *A. baumannii* seems be due to different strains carrying Tn*125* or derivatives of Tn*125* rather than plasmid-mediated or clonal (Bonnin et al., 2013; Poirel et al., 2012a).

## CARBAPENEM-HYDROLYZING CLASS D β-LACTAMASES

Oxacillinases comprise a heterogeneous group of class D β-lactamases which are able to hydrolyze amino- and carboxypenicillins (Poirel et al., 2010b). The majority of class D β-lactamases are not inhibited by commercially available β-lactamase inhibitors but are inhibited *in vitro* by NaCl. Over 250 types of oxacillinases are reported with a minority demonstrating low levels of carbapenem-hydrolyzing activity. This select group of enzymes is also referred to as the carbapenem-hydrolyzing class D β-lactamases (CHDLs; **Table 3**). CHDLs have been identified most frequently in *Acinetobacter* spp., however, there has been increasing isolation among Enterobacteriaceae, specifically OXA-48 (oxacillinase-48; Lascols et al., 2012; Mathers et al., 2012).

**Table 3:** Carbapenem-hydrolyzing class D β-lactamases.

Enzyme group	Year isolated or described	Organism(s)	Geographic distribution	Location	Reference
OXA-23/27	1985/–****	*Acinetobacter baumannii*, Proteus mirabilis*	Europe, USA, Middle East, Asia, Australia	Plasmid, chromosomal	Afzal-Shah et al. (2001), Gogou et al. (2011)
OXA-24/40	1997	*A. baumannii*	Europe and USA	Plasmid, chromosomal	Bou et al. (2000b), Lopez-Otsoa et al. (2002)
OXA-25	–	*A. baumannii*	Spain	Chromosomal	Afzal-Shah et al. (2001)
OXA-26	1996	*A. baumannii*	Belgium	Chromosomal	Afzal-Shah et al. (2001)
OXA-48	2001	*K. pneumoniae*, Enterobacteriaceae	Turkey, Middle East, Northern Africa, Europe, India, USA	Plasmid	Poirel et al. (2004b)
OXA-51/66/69	1993	*A. baumannii*	Worldwide	Chromosomal	Brown et al. (2005), Evans et al. (2007)
OXA-58	2003	*A. baumannii*	Europe, USA, Middle East, South America	Plasmid	Poirel et al. (2005)
OXA-143	2004	*A. baumannii*	Brazil	Plasmid	Higgins et al. (2009)
OXA-162	2008	Enterobacteriaceae	Germany	Plasmid	Pfeifer et al. (2012)
OXA-163	2008	*K. pneumoniae*, *E. coli*	Argentina and Egypt	Plasmid	Poirel et al. (2011b), Abdelaziz et al. (2012)
OXA-181	2006	*K. pneumoniae*, *E. coli*	India	Plasmid	Castanheira et al. (2011)
OXA-204	2012	*K. pneumoniae*	Tunisia	Plasmid	Potron et al. (2013)
OXA-232	2012	*K. pneumoniae*	France	Plasmid	Poirel et al. (2012c)

* Single isolate described in France.

With the exception of OXA-163 (Poirel et al., 2011b), CHDLs efficiently inactivate penicillins, first generations cephalosporins, and β-lactam/β-lactamase inhibitor combinations, but spare extended-spectrum cephalosporins. Carbapenem hydrolysis efficiency is lower than that of other carbapenemases, including the MBLs, and often additional resistance mechanisms are expressed in organisms demonstrating higher levels of phenotypic carbapenem resistance. These include expression of other carbapenemases, alterations in outer membrane proteins (e.g., CarO, OmpK36; Perez et al., 2007; Gülmez et al., 2008; Pfeifer et al., 2012), increased transcription mediated by *IS* elements functioning as promoters, increased gene copy number, and amplified drug efflux (Poirel and Nordmann, 2006; Perez et al., 2007). Many subgroups of CHDLs have been described. We will focus on those found in *A. baumannii* and Enterobacteriaceae: OXA-23 and OXA-27; OXA-24/40, OXA-25, and OXA-26; OXA-48 variants; OXA-51, OXA-66, OXA-69; OXA-58, and OXA-143.

CHDLs can be intrinsic or acquired. *A. baumannii* does have naturally occurring but variably expressed chromosomal CHDLs, OXA-51, OXA-66, and OXA-69 (Brown et al., 2005; Héritier et al., 2005b). For the most part, in isolation the phenotypic carbapenem resistance associated with these oxacillinases is low. However, levels of carbapenem resistance appear to be increased in the presence of specific insertion sequences promoting gene expression (Figueiredo et al., 2009; Culebras et al., 2010). Additional resistance to extended-spectrum cephalosporins can be seen in the setting of co-expression of ESBLs and/or other carbapenemases (Castanheira et al., 2011; Mathers et al., 2012; Pfeifer et al., 2012; Voulgari et al., 2012; Potron et al., 2013).

The first reported “acquired” oxacillinase with appreciable carbapenem-hydrolyzing activity was OXA-23. OXA-23, or ARI-1, was identified from an *A. baumannii* isolate in Scotland in 1993 (the isolate was first recovered in 1985; Paton et al., 1993). Subsequently, OXA-23 expression has been reported worldwide (Mugnier et al., 2010) and both plasmid and chromosomal carriage of *bla*_OXA-23_ are described. The OXA-23 group includes OXA-27, found in a single *A. baumannii* isolate from Singapore (Afzal-Shah et al., 2001). With the exception of an isolate of *Proteus mirabilis* identified in France in 2002, this group of β-lactamases has been exclusively recovered from *Acinetobacter* species (Bonnet et al., 2002). Increased expression of OXA-23 has been associated with the presence of upstream insertion sequences (e.g., IS*Aba1* and IS*Aba4*) acting as strong promoters (Corvec et al., 2007).

Another group of CHDLs include OXA-24/40, OXA-25, and OXA-26 (Bou et al., 2000b; Afzal-Shah et al., 2001). OXA-24 and OXA-40 differ by a few amino acid substitutions and OXA-25 and OXA-26 are point mutation derivatives of OXA-40 (Afzal-Shah et al., 2001). Although primarily linked with clonal outbreaks in Spain and Portugal (Bou et al., 2000a; Lopez-Otsoa et al., 2002; Da Silva et al., 2004; Acosta et al., 2011), OXA-24/40 β-lactamases has been isolated in other European countries and the USA (Lolans et al., 2006). OXA-40 was in fact the first CHDL documented in the USA (Lolans et al., 2006).

OXA-58 has also only been detected in *Acinetobacter* spp. initially identified in France (Héritier et al., 2005a; Poirel et al., 2005), OXA-58 has been associated with institutional outbreaks and has been recovered from clinical isolates of *A. baumannii* worldwide (Coelho et al., 2006; Mendes et al., 2009; Gales et al., 2012).

As civilian and military personnel began returning from Afghanistan and the Middle East, practitioners noted increasing recovery of *A. baumannii* from skin and soft tissue infections. Drug resistance was associated with expression of both OXA-23 and OXA-58 (Hujer et al., 2006; Scott et al., 2007; Perez et al., 2010b). Many isolates carrying the *bla*_OXA-58_ gene concurrently carry insertion sequences (e.g., IS*aba1*, IS*Aba2*, or IS*Aba3*) associated with increased carbapenemase production and thus higher levels of carbapenem resistance. In one report increased gene copy number was also associated with a higher level of enzyme production and increased phenotypic carbapenem resistance (Bertini et al., 2007).

Spread of OXA-type carbapenemases among *A. baumannii* appears to be clonal and in depth reviews of the molecular epidemiology and successful dissemination of these clones have been published (Woodford et al., 2011; Zarrilli et al., 2013). Two MLST schemes with three loci in common exist for *A. baumannii* – the PubMLST scheme (Bartual et al., 2005) and the Pasteur scheme (Diancourt et al., 2010). Both schemes assign different sequence types into clonal complexes (CC). Sequence types and CC from both schemes can be further categorized into the international (European) clones I, II, and III. It should be noted, however, that the molecular taxonomy of *A. baumannii* continues to evolve (Higgins et al., 2012a). OXA-23 producing *A. baumannii* predominantly belong to international clones I and II with a notable proportion being part of CC92 (PubMLST; Mugnier et al., 2010; Adams-Haduch et al., 2011). Similarly, *A. baumannii* isolates associated with epidemic spread of OXA-24/40 in Portugal and Spain appear are incorporated in international clone II (Da Silva et al., 2004; Grosso et al., 2011) and ST 56 (PubMLST; Acosta et al., 2011). OXA-58 expressing *A. baumannii* have been associated with international clones I, II, and II and a variety of unrelated sequence types (Di Popolo et al., 2011; Gogou et al., 2011).

OXA-48 was originally identified in a carbapenem-resistant isolate of *K. pneumoniae* in Turkey (Poirel et al., 2004c). Early reports suggested that this enzyme was geographically restricted to Turkey. In the past few years, however, the enzyme has been recovered from variety of Enterobacteriaceae and has successfully circulated outside of Turkey with reports of isolation in the Middle East, North Africa, Europe (Carrer et al., 2010), and most recently the USA (Lascols et al., 2012; Mathers et al., 2012). The Middle East and North Africa may be secondary reservoirs for these CHDLs (Hays et al., 2012; Poirel et al., 2012c). Indeed, the introduction of OXA-48 expressing Enterobacteriaceae in some countries has been from patients from the Middle East or Northern Africa (Decre et al., 2010; Adler et al., 2011; Poirel et al., 2011e; Canton et al., 2012). In the USA, the first clinical cases were associated with ST 199 and ST 43 (Mathers et al., 2012).

At least six OXA-48 variants (e.g., OXA-48, OXA-162, OXA-163, OXA-181, OXA-204, and OXA-232) have been identified. OXA-48 is by far the most globally dispersed and its epidemiology has been recently reviewed (Poirel et al., 2012c). Unlike KPCs and NDM-1 which have been associated with a variety of plasmids, a single 62 kb self-conjugative IncL/M-type plasmid has contributed to a large proportion of the distribution of *bla*_OXA-48_ in Europe (Potron et al., 2011a). Sequencing of this plasmid (pOXA-48a) notes that *bla*_OXA-48_ had been integrated through the acquisition of a Tn*1999* composite transposon (**Figure 1C**; Poirel et al., 2012b) *bla*_OXA-48_ appears to be associated with a specific insertion sequence, IS*1999* (Poirel et al., 2004c, 2012b). A variant of Tn*1999*, Tn*1999.2*, has been identified among isolates from Turkey and in Europe (Carrer et al., 2010; Potron et al., 2011a). Tn*1999.2* harbors an IS*1R* element within the IS*1999*. OXA-48 appears to have the highest affinity for imipenem of the CHDLs specifically those harboring *bla*_OXA-48_ within a Tn*1999.2* composite transposon (Docquier et al., 2009). Three isoforms of the Tn*1999* transposon have been described (Giani et al., 2012).

Although much of the spread of OXA-48 is attributed to a specific plasmid, outbreak evaluations demonstrate that a variety of strains have contributed to dissemination of this emerging carbapenemase in *K. pneumoniae*. The same *K. pneumoniae* sequence type, ST 395, harboring *bla*_OXA-48_ was identified in Morocco, France, and the Netherlands (Cuzon et al., 2011; Potron et al., 2011a). ST 353 was associated with an outbreak of OXA-48 producing *K. pneumoniae* in London (Woodford et al., 2011) and ST 221 with an outbreak of OXA-48 in Ireland (Canton et al., 2012). OXA-48 production in *K. pneumoniae*, like KPC-expressing *K. pneumoniae*, has also been associated with ST 14 (Poirel et al., 2004c) and a recent outbreak in Greece was associated with ST 11 (Voulgari et al., 2012).

*bla*_OXA-48_ is remarkably similar to *bla*_OXA-54_, a β-lactamase gene intrinsic to *Shewanella oneidensis* (Poirel et al., 2004a). *Shewanella* spp. are relatively ubiquitous waterborne Gram-negative bacilli and are proving to be a potential environmental reservoir for OXA-48 like carbapenemases as well as other resistance determinants (Héritier et al., 2004; Poirel et al., 2004b; Potron et al., 2011b).

OXA-163, a single amino acid variant of OXA-48, was identified in isolates of *K. pneumoniae* and *Enterobacter cloacae* from Argentina and is unique in that it has activity against extended-spectrum cephalosporins (Poirel et al., 2011b). OXA-163 also has been identified in Egypt, which has a relatively prevalence of OXA-48, in patients without epidemiologic links to Argentina (Abdelaziz et al., 2012).

OXA-181 was initially identified among carbapenem-resistant Enterobacteriaceae collected from India (Castanheira et al., 2011). OXA-181 differs from OXA-48 by four amino acids, however, appears to be nestled in an entirely different genetic platform. The *bla*_OXA-181_ gene has been mapped to a different group of plasmids, the ColE family, and has been associated with an alternative insertion sequence, IS*Ecp1*. The latter insertion sequence has been associated with the acquisition of other β-lactamases including CTX-M-like ESBLs. Like, OXA-48, it appears that OXA-181 may have evolved from a waterborne environmental species *Shewanella xiamenensis* (Potron et al., 2011b).

OXA-204 differs from OXA-48 by a two amino acid substitution. It was recently identified in a clinical *K. pneumoniae* isolate from Tunisia (Potron et al., 2013). Its genetic construct appears to be similar to that of OXA-181. OXA-232 was recently identified among *K. pneumoniae* isolates in France (Poirel et al., 2012c).

OXA-143 is a novel plasmid-borne carbapenem-hydrolyzing oxacillinase recovered from clinical *A. baumannii* isolates in Brazil (Higgins et al., 2009). Information regarding its significance and prevalence continues to evolve (Antonio et al., 2010; Werneck et al., 2011; Mostachio et al., 2012).

## AVAILABLE AGENTS AND DRUGS IN DEVELOPMENT

Few antimicrobials are currently available to treat infections with carbapenemase-producing Gram-negative bacteria. Carriage of concurrent resistance determinants can result in decreased susceptibility non-β-lactams including the fluoroquinolones and aminoglycosides thus further compromising an already limited antimicrobial arsenal. What frequently remains available are the polymyxins (including colistin), tigecycline, and fosfomycin but susceptibilities to these agents are unpredictable (Falagas et al., 2011).

The reintroduction of polymyxins, both polymyxin B and colistin overlaps with the evolution of carbapenem resistance among Gram-negative bacilli. The clinical “resurgence” of these agents is well documented (Falagas and Kasiakou, 2005; Li et al., 2006a; Landman et al., 2008). Some experts advocate for the use of polymyxins in combination with other agents like rifampicin (Hirsch and Tam, 2010; Urban et al., 2010). *In vitro* evaluations of different combinations including carbapenems, rifamycins, and/or tigecycline demonstrate variable results (Bercot et al., 2011; Biswas et al., 2012; Deris et al., 2012; Jernigan et al., 2012). Most evaluations of the clinical outcomes or “effectiveness” of combination therapies have been retrospective (Qureshi et al., 2012; Tumbarello et al., 2012). Prospective clinical trials evaluating the superiority of colistin-based combination therapy over monotherapy are in their infancy. A real interest in combination therapy persists due to the concern of hetero-resistance (Li et al., 2006b; Poudyal et al., 2008; Lee et al., 2009; Yau et al., 2009; Meletis et al., 2011).

Early evaluations of the glycylcycline, tigecycline, demonstrated favorable *in vitro* activity against ESBL-producing Enterobacteriaceae and specific isolates of carbapenem-resistant *A. baumannii* and Enterobacteriaceae (Bratu et al., 2005; Fritsche et al., 2005; Noskin, 2005; Castanheira et al., 2008; Wang and Dowzicky, 2010). Tigecycline remains untested in prospective trials and reports of resistance are increasing (Navon-Venezia et al., 2007; Anthony et al., 2008; Wang and Dowzicky, 2010; Sun et al., 2012). The role of tigecycline in treating primary bloodstream infections or urinary tract infections remains undefined due less than therapeutic concentrations of drug achieved in the serum (Rodvold et al., 2006) and urine (Satlin et al., 2011). We also note that meta-analyses of pooled data from trials evaluating the use of tigecycline for a variety of indications suggest there is a excess mortality associated with the use of tigecycline over comparator regimens (Cai et al., 2011; Tasina et al., 2011; Yahav et al., 2011; Verde and Curcio, 2012). However, in the absence of other tested regimens tigecycline may be an appropriate or perhaps the only therapeutic option.

Growing resistance to both the polymyxins and tigecycline has resulted the revisiting of older drugs including chloramphenicol, nitrofurantoin, and temocillin (Livermore et al., 2011d). Fosfomycin is also one of these earlier antibiotics being reassessed (Falagas et al., 2008). In an *in vitro* evaluation of 68 KPC-expressing *K. pneumoniae* isolates, fosfomycin demonstrated *in vitro* activity against 87% of tigecycline and/or polymyxin non-susceptible isolates and 83% of isolates that were resistant to both (Endimiani et al., 2010b). Fosfomycin may be a potential therapeutic option for patients infected with carbapenemase-producing Enterobacteriaceae if the infection is localized to the genitourinary tract. Unfortunately, fosfomycin does not demonstrate reliable activity against non-urinary pathogens. Fosfomycin demonstrated activity against only 30.2% of 1693 multidrug-resistant (MDR) *P. aeruginosa* isolates and 3.5% of 85 MDR *A. baumannii* isolates (Falagas et al., 2009). The individual studies included in this review did not employ uniform MDR definitions or consistent susceptibility breakpoints. Moreover, access to the parenteral fosfomycin is limited and the threshold for resistance is low (Rodriguez-Rojas et al., 2010; Karageorgopoulos et al., 2012). Concerns regarding the emergence of resistance have lead to an increasing interest in the utility of combination therapy (Michalopoulos et al., 2010; Bercot et al., 2011; Souli et al., 2011).

Few agents are in the advanced stages of development with demonstrable *in vitro* activity against carbapenemase-producing organisms. These include β-lactamase inhibitors, aminoglycoside derivatives, polymyxin derivatives, and novel monobactams and monobactams-β-lactamase inhibitor combinations.

Avibactam, or NXL104, is a β-lactamase inhibitor which has been tested in combination with ceftazidime, ceftaroline, and aztreonam against several carbapenemase-producing Enterobacteriaceae with impressive decreases in MICs (Livermore et al., 2008, 2011b; Endimiani et al., 2009a; Mushtaq et al., 2010c). Cephalosporin–avibactam combinations do not inhibit MBLs. Avibactam in combination with aztreonam, however, does seem to demonstrate activity against isolates harboring a variety of carbapenem resistance mechanisms including MBLs (Livermore et al., 2011b). Regrettably, the avibactam and aztreonam combination is not currently in clinical trials. The combination of ceftazidime–avibactam has been evaluated against collections of non-fermenting Gram-negative pathogens and its role remains undefined (Mushtaq et al., 2010b). In some evaluations of ceftazidime non-susceptible isolates of *P. aeruginosa* decrease MICs were noted with the addition of avibactam (Mushtaq et al., 2010b; Walkty et al., 2011; Crandon et al., 2012; Levasseur et al., 2012). The combinations of ceftaroline–avibactam and ceftazidime–avibactam are currently in clinical trials.

Methylidene penems (penem-1 and penem-2) are β-lactamase inhibitors and appear to be potent inhibitors of KPC-2 (Papp-Wallace et al., 2010). The combination of cefepime with penem-1 demonstrated lower cefepime MICs in 88.1% of the 42 KPC-producing *K. pneumoniae* isolates evaluated (Endimiani et al., 2010a). MK-7655 is a novel β-lactamase being evaluated in combination with imipenem against carbapenem-resistant Gram-negative bacilli (Hirsch et al., 2012).

ME1071, formerly CP3242 (Bassetti et al., 2011), is a maleic acid derivative that competitively inhibits MBLs. Earlier studies demonstrated concentration-dependent decreases in carbapenem MICs in MBL-producing *P. aeruginosa* (Ishii et al., 2010), *A. baumannii*, and select Enterobacteriaceae (Shahid et al., 2009) A contemporary pre-clinical evaluation of ME1071 in combination with various type 2 carbapenems (i.e., biapenem, doripenem, meropenem, imipenem) confirms remarkable decreases in the carbapenem MICs for Enterobacteriaceae and *A. baumannii* harboring IMP, VIM, and NDM-type MBLs (Livermore et al., 2013). Irrespective of the candidate carbapenem, ME1071 activity against NDM MBLs was less than that of VIM-type and IMP-type MBLs. Of note, biapenem was the carbapenem with the lowest baseline MICs to the MBLs, but it is commercially unavailable in many countries including the USA. Other MBL-specific inhibitors are in pre-clinical development (Chen et al., 2012).

Plazomicin (ACHN-490) is an aminoglycoside derivative with potent activity against some carbapenem-resistant Gram-negative bacilli (Zhanel et al., 2012). Studies have noted that susceptibilities to aminoglycosides vary among KPC-producing *K. pneumoniae*. In one evaluation, 48% of 25 tested isolates were susceptible to amikacin, 44% to gentamicin, and 8% to tobramycin. Plazomicin demonstrated an MIC_90_ significantly lower than that of amikacin (Endimiani et al., 2009c). *In vitro* studies also indicate that depending on the aminoglycoside resistance mechanisms present, Plazomicin may have activity against select isolates of *P. aeruginosa* and *A. baumannii* (Aggen et al., 2010; Landman et al., 2011). Susceptibility to plazomicin in the setting of resistance to other aminoglycosides appears to be dependent on the mechanism of aminoglycoside resistance (Livermore et al., 2011a).

NAB739 and NAB7061 are polymyxin derivatives that may be less nephrotoxic than commercially available polymyxins. In a small *in vitro* study, NAB739 displayed activity against nine carbapenemase-producing polymyxin-susceptible isolates of Enterobacteriaceae (Vaara et al., 2010). A contemporary evaluation of NAB739 demonstrated higher MICs compared to those of polymyxin B in a collection of polymyxin-susceptible and non-susceptible Enterobacteriaceae, *P. aeruginosa*, and *A. baumannii* (Vaara et al., 2012). NAB7061 when used in combination with rifampicin or clarithromycin demonstrated synergistic activity against seven strains of carbapenemase-producing Gram-negative bacilli including one polymyxin-resistant strain (Vaara et al., 2010). It remains unclear what role these agents will play in the setting the increasing burden of infections with carbapenemase-producing Enterobacteriaceae.

The activity of the siderophore monosulfactam, BAL30072, has been against non-fermenting carbapenemase-producing Gram-negative bacilli (Page et al., 2010). In one study, susceptibility to BAL30072 was noted in 73% of 200 isolates of carbapenemase-producing *A. baumannii*, the majority of which were of the same OXA-23 producing clone (Mushtaq et al., 2010a). In that same study, smaller percentages of susceptibility were noted in a selection of carbapenem-resistant *Burkholderia cepacia* and *P. aeruginosa* isolates. Recent evaluations of BAL30072 confirm that there may be a role for this agent in the treatment of resistant *A. baumannii* infections (Russo et al., 2011; Higgins et al., 2012b). BAL 30376 is a combination of a siderophore monobactam with clavulanic acid. In two studies, this combination demonstrated reasonable *in vitro* activity against CHDL, including OXA-48, and MBLs but not KPCs (Livermore et al., 2010; Page et al., 2011).

## CONCLUDING REMARKS

In the last 5 years, we have witnessed the global spread of carbapenem resistance among Gram-negative organisms. The notion that multidrug resistance among these pathogens is limited to isolated outbreaks among the critically ill has met the ultimate challenge with NDM-1 (Kumarasamy et al., 2010). The conveniences of travel and medical tourism have introduced resistance mechanisms across states, countries, and even continents at an alarming rate (Rogers et al., 2011; van der Bij and Pitout, 2012). Rates of resistance in some countries may be underestimated due to the lack of organized reporting structures and limited resources. Long-term healthcare facilities are now recognized reservoirs for the continued propagation of MDR organisms (Urban et al., 2008; Aschbacher et al., 2010; Perez et al., 2010a; Ben-David et al., 2011; Prabaker et al., 2012; Viau et al., 2012).

Until the introduction of accurate, affordable, and readily accessible diagnostics and reliably effective antimicrobials a major focus remains containment and eradication of these organisms within the healthcare environment. Many cite a “bundle” type approach that includes administrative support, active surveillance, antimicrobial stewardship, and augmented infection control practices (Centers for Disease Control and Prevention, 2009; Schwaber et al., 2011; Snitkin et al., 2012). Just as with drug development (Tillotson, 2010), the future savings of investing in prevention is not as tangible as the immediate capital investment required to allot appropriate resources including advanced laboratory platforms, experienced laboratory personnel, dedicated nursing staff, and infection control personnel (Bilavsky et al., 2010). Expanding these efforts to non-acute healthcare settings is recommended to begin to stem the evolving pandemic of carbapenem resistance (Gupta et al., 2011).

The prudent use of antibiotics is essential in combating the continuing evolution of resistance (Marchaim et al., 2012). This may be even more crucial in areas where non-prescription antimicrobial use is common and continues to be unregulated. In an age where multidrug resistance is so widespread, even the appropriate use of broad-spectrum antibiotics has contributed to our current state.

Research funding and support for the description of resistance mechanisms, validation of current infection control practices, and antimicrobial development must be prioritized. Institutions supporting infection control, state of the art microbiology laboratories, and antimicrobial stewardship programs should receive recognition and incentives for their foresight. Despite these continuing challenges, considerable progress has been made to identify at-risk populations and to describe resistance determinants. Collaborative efforts (Kitchel et al., 2009a; Struelens et al., 2010; Canton et al., 2012) have led to a better understanding and awareness of the epidemiology and the contribution of antimicrobial use and the environment to the propagation of antimicrobial resistance. These joint efforts have proven crucial for the propagation of information about carbapenemases. Continuing to encourage these partnerships is imperative in the ongoing struggle against antimicrobial resistance and to prevent antimicrobials from essentially becoming obsolete.

## Conflict of Interest Statement

The authors declare that the research was conducted in the absence of any commercial or financial relationships that could be construed as a potential conflict of interest.
